# SARS-CoV-2 Seropositivity in Asymptomatic Frontline Health Workers in Ibadan, Nigeria

**DOI:** 10.4269/ajtmh.20-1235

**Published:** 2020-11-11

**Authors:** Olatunde Olayanju, Olabisi Bamidele, Fabian Edem, Bola Eseile, Abimbola Amoo, Jude Nwaokenye, Chioma Udeh, Gabriel Oluwole, Gabriel Odok, Nnaemeka Awah

**Affiliations:** 1 Chemical Pathology Department, University College Hospital, Ibadan, Nigeria;; 2 Medicine Department, University of Cape Town, Cape Town, South Africa;; 3 Immunology Department, University College Hospital, Ibadan, Nigeria;; 4 Medical Microbiology Department, University College Hospital, Ibadan, Nigeria;; 5 Medicine Department, University College Hospital, Ibadan, Nigeria

## Abstract

Global health has been thrown into turmoil by the COVID-19 pandemic, which has caused devastating morbidity and unprecedented loss of life in almost all continents of the world. It was predicted that the magnitude of the pandemic in Africa will be high because of poor health structure and intensely poor living condition, but that has not happened, surprisingly. It was hypothesized that the youthful population and a vastly primed immune system were protective, and many people may have been exposed without coming down with the severe disease. Most of them would have presented in hospitals with other medical conditions and possibly transmit COVID-19 to health workers inadvertently. This study is designed to measure serum SARS-CoV-2 IgG levels in health workers as a marker of latent exposure. Asymptomatic frontline health workers were randomly selected from the University College Hospital Ibadan, Nigeria; venous blood samples were obtained from them, and the serum SARS-CoV-2 IgG level was determined using ELISA techniques. A proportion of participants with seropositivity were obtained, and factors associated with seropositivity were determined. A total of 133 participants were recruited for this study, and 60 (45.1%) of them were seropositive for SARS-CoV-2. Among the seropositive participants were doctors, nurses, health assistants, laboratory scientists and technicians, and nonmedical staff. Obstetrics, gynecology, and emergency departments had higher odds of seropositivity. Seroprevalence of SARS-CoV-2 is very high among frontline health workers, though asymptomatic. This calls for a more stringent precaution against further spread within the hospital environment.

## INTRODUCTION

COVID-19 became a pandemic, ravaging the whole world and constituting a huge threat to global health.^1–3^ More than 200 countries and territories of the world have reported cases running into more than 30 million with a mortality of more than 945,000 by mid-September 2020.^4^ The United States is the worst hit with more than six million cases, and several other countries like India, Brazil, the United Kingdom, and Mexico have reported more than 40,000 deaths each.^4^ In Nigeria, about 60,000 cases have been reported with close to 1000 mortalities around the same period. All of these countries have instituted several measures to curtail the spread of the virus, although the number of cases is rising in some countries, flattening in others, while some are experiencing a decline.

The WHO and several other stakeholders warned that the effect of the pandemic will be devastating in Africa because of weak health systems, inadequate health infrastructure, and the colossal poverty status of most countries on the continent.^5–7^ However, the dynamics of the disease in Africa have not only surprised the world but also have defied all predictions from physicians, epidemiologists, and scientists globally.^8,9^ This is despite poor adherence to social distancing rule, overcrowded markets, and living homes.^10^ Although the WHO suggested that the low cases were due to low testing rates across the continent, there has been no increase in clinical cases suggestive of the disease or reports of unexplainable deaths which could justify the WHO’s stance.^11,12^

Several hypotheses were put forward to explain the peculiarity of the disease in Africa. These include a predominantly young population, high humidity, and protective cross-immunity from the myriads of endemic communicable diseases which may have primed the immune system.^10,13^ It is believed that most Africans have been exposed to the virus but did not come down with a severe illness because of these reasons. Many patients have presented to different hospitals for totally unrelated conditions and may have transmitted the infection to health workers without knowing. In fact, so many health workers, though asymptomatic, have tested positive for the virus suggesting that many more may have been through the completed life cycle of the virus incognito. To examine this claim, this study is designed to detect SARS-CoV-2 viral IgG antibody in the serum of frontline healthcare workers at the University College Hospital, a tertiary hospital with 850 beds in Ibadan, Nigeria.

## METHODS

### Participants.

This is a hospital-based cross-sectional study; healthcare workers who had not taken the COVID-19 test and had no COVID-19–related symptoms were randomly selected from different departments of the hospital. A structured questionnaire was administered to every participant to obtain information about sociodemographic, medical, and travel history. Some of the required information were age, gender, occupation, travel history between December 2019 and April 2020, comorbid condition, and involvement in the care for COVID-19 patients. Participants with recent febrile or respiratory illnesses were excluded from the study. Ethical approval was obtained from the University of Ibadan/University College Hospital Research Ethics Committee.

### Sample collection and processing.

About 2 mL of venous blood was obtained from the participants and stored in plain sample bottles; these were left to clot while standing for 2 hours at room temperature. The clotted samples were centrifuged at 1000 × *g* for 20 minutes, and the sera obtained were frozen and stored at −20°C until the time of analysis which lasted about 1 month. Samples were analyzed using the ELISA technique for qualitative SARS-CoV-2 spike protein IgG according to the manufacturer’s protocol (Elabscience Biotechnology Inc., Houston, TX). The manufacturer’s declared intra-assay and inter-assay coefficients of variations were < 8% and < 10%, respectively, on low-, mid-, and high-level samples, each being tested 20 times.

### Statistical analysis.

Qualitative and quantitative variables including the proportion of seropositivity were reported in percentage. Univariate logistic regression was used to determine the relationship between demographic and clinical variable and seropositivity to the SARS-CoV-2 IgG. A *P*-value of < 0.05 was taken as statistically significant. Analysis was carried out using Statistical Package for Social Sciences (SPSS) version 26.0 (IBM Inc., Armonk, NY).

## RESULTS

A total of 133 hospital workers were recruited for this study. They comprised 55 (41.4%) medical doctors, 33 nurses (24.8%), 19 (14.3%) health assistants, 18 (13.5%) laboratory scientists and technicians, and eight (6.0%) nonmedical staff working in various departments within the hospital (Table 1). Overall, 63 (47.4%) of the participants were male, and the departments from where recruitment was carried out included surgery, medicine, emergency, private suite, obstetrics and gynecology (O&G), chemical pathology, hematology, microbiology, and accounts (Table 1). Close to half of the entire participants (49%) were between the age of 31 and 40 years.

**Table 1:** Sociodemographic characteristics and SARS-CoV-2 IgG seroprevalence among participants in this study

Variable	Category	Frequency
Age range (years)	20–30	36 (27.1)
	31–40	65 (48.9)
	41–50	26 (19.6)
	51–60	6 (4.5)
Gender	Male	63 (47.4)
	Female	70 (52.6)
Department	Surgery	13 (9.8)
	Medicine	17 (12.8)
	Emergency	42 (31.6)
	Private suite	14 (10.5)
	Obstetrics and gynecology	10 (7.5)
	Chemical pathology	22 (16.5)
	Hematology	8 (6.0)
	Microbiology	6 (4.5)
	Accounts	1 (0.8)
Occupation	Doctor	55 (41.4)
	Nurse	33 (24.8)
	Health assistant	19 (14.3)
	Laboratory scientist/technician	18 (13.5)
	Nonmedical staff	8 (6.0)
Serum SARS-CoV-2 IgG	Negative	73 (54.9)
	Positive	60 (45.1)

Data are presented as number (percentage).

Sixty participants were positive for SARS-CoV-2 IgG, indicating a seroprevalence of 45.1% in the study population (Table 1). Among the seropositive participants were 27 (45.0%) doctors, 14 (23.3%) nurses, 10 (16.7%) health assistants, four (6.7%) laboratory scientists and technicians, and five (8.3%) nonmedical staff (Table 2). Grouping of study participants by department where they work showed that 90% of O&G staff, 60% of emergency staff, 59% of medicine staff, 33% of microbiology staff, 32% of chemical pathology staff, 23% of surgery staff, and 21% of private suite staff recruited for this study were seropositive for SARS-CoV-2, whereas none of the study participants recruited from hematology was seropositive.

**Table 2:** Logistic regression showing the relationship between demographic and clinical information and SARS-CoV-2 IgG seropositivity

Variable	Category	Positive (*n* = 60)	Negative (*n* = 73)	OR	95% CI	*P*-value
Age range (years)	21–30	14 (23.3)	22 (30.6)	–	–	–
	31–40	31 (51.7)	33 (45.8)	1.429	0.621–3.285	0.401
	41–50	14 (23.3)	12 (16.7)	1.833	0.660–5.092	0.245
	51–60	1 (1.7)	5 (6.9)	0.314	0.033–2.979	0.186
Gender	Male	32 (54.2)	29 (40.3)	0.587	0.292–1.181	0.135
	Female	27 (45.8)	43 (59.7)	–	–	–
Department	Chemical pathology	7 (11.7)	15 (20.5)	–	–	–
	Surgery	3 (5.0)	10 (13.7)	0.643	0.134–3.095	0.582
	Medicine	10 (16.7)	7 (9.6)	3.061	0.819–11.440	0.096
	Emergency	25 (41.7)	17 (23.3)	3.151	1.061–9.357	0.039*
	Private suite	3 (5.0)	11 (15.1)	0.584	0.123–2.782	0.500
	Obstetrics and gynecology	9 (15.0)	1 (1.4)	19.286	2.028–183.412	0.010*
	Hematology	0 (0.0)	8 (11.0)	0.000	0.000	0.999
	Microbiology	2 (3.3)	4 (5.5)	1.071	0.157–7.307	0.944
	Accounts	1 (1.7)	0 (0.0)	N/A	N/A	N/A
Occupation	Nonmedical	5 (8.3)	3 (4.1)	–	–	–
	Doctor	27 (45.0)	28 (38.4)	0.929	0.122–7.080	0.943
	Nurse	14 (23.3)	19 (26.0)	0.778	0.097–6.230	0.813
	Health assistant	10 (16.7)	9 (12.3)	0.714	0.086–5.959	0.756
	Laboratory scientist/technologist	4 (6.7)	14 (19.2)	0.444	0.045–4.374	0.487

N/A = not applicable; OR = odds ratio. Data are presented as number (percentage).

* Indicates statistically significant.

Logistic regression analysis showed no association between seropositivity and participants’ age, gender, or occupation (*P* > 0.05) but was associated with the participants’ departments. The participants from emergency and O&G departments showed significantly higher odds of seropositivity (odds ratio [OR] = 3.151; *P* = 0.039 and OR = 19.286; *P* = 0.010, respectively; Table 2). History of travels within Nigeria during the pandemic (OR = 1.329; *P* = 0.428) and being in a gathering of more than 100 people (OR = 1.102; *P* = 0.790) were associated with increased risk of seropositivity, though not statistically significant (Table 3).

**Table 3:** Logistic regression showing the relationship between travel history and self-reported contact with COVID-19 patients and SARS-CoV-2 IgG seropositivity

Variable	Category	Positive	Negative	OR	95% CI	*P*-value
Travel outside Nigeria	Yes	1 (1.7)	3 (4.1)	0.409	0.041–4.042	0.445
	No	58 (98.3)	70 (95.9)			
Travel within Nigeria	Yes	25 (41.7)	26 (35.6)	1.329	0.657–2.688	0.428
	No	35 (58.3)	47 (64.4)			
Contact with person(s) who traveled within or outside Nigeria	Yes	35 (58.3)	49 (67.1)	0.714	0.350–1.457	0.355
	No	25 (41.7)	24 (32.9)			
Presence in the gathering of > 100 people	Yes	22 (38.6)	27 (37.0)	1.102	0.538–2.257	0.790
	No	35 (61.4)	46 (63.0)			
Contact with COVID-19 case	Yes	41 (71.9)	55 (76.4)	0.845	0.378–1.887	0.681
	No	16 (28.1)	17 (23.6)			

OR = odds ratio.

## DISCUSSION

The impact of the COVID-19 pandemic in Africa did not reflect the devastating prediction from the WHO and other health agencies across the world. Although the magnitude of morbidity among healthcare workers was quite high in most countries with high disease prevalence, the same cannot be said for Africa, despite low precautionary measures.^14^ In this study, 45% of healthcare workers were seropositive for SARS-CoV-2, despite having no symptoms and providing routine services to patients presenting with a myriad of clinical conditions. Seropositivity was found across all cadres of healthcare workers, ranging from clinical personnel to nonmedical staff members. Workers within the age of 31–40 years were mostly affected, and those from the O&G and emergency departments recorded the highest odds of seropositivity.

The prevalence of COVID-19 is believed to be grossly underreported across the globe^15^; several countries have gone ahead to publish seroprevalence among the general population.^16–18^ Disease burden in Africa was predicted to outrightly overwhelm the already weak health system with a high mortality rate far above the European and the American figures.^19,20^ However, only five African countries (South Africa, Egypt, Morocco, Algeria, and Nigeria) are in the top 50 countries with the highest mortality rate worldwide.^21^ There is no evidence to support that Africa did not have a good share of the pandemic, given the volume of trade and travel between the continent and Asia, Europe, and America. There is however a good reason to believe that Africa did not experience the same severity of disease found in the other continents.^22^ Objectively, the seroprevalence of COVID-19 among asymptomatic healthcare workers in this study was 45%, indicating a latent exposure to the disease, and this is even higher than seroprevalence in the general population in France, Italy, and Spain where mortality was massive.^23^

This high prevalence is not surprising because the hospitals were opened for most of the pandemic period, and healthcare workers continued to attend patients with minimal precautionary measures. Most of the patients presented with conditions mostly unrelated to COVID-19, whereas those with risk factors including travel history and contact with COVID-19 patients deliberately refused to disclose their status in the hospitals for fear of stigmatization.^24^ This attitude is believed to have encouraged a covert transmission among the general population and, consequently, the healthcare workers.^24^ Surprisingly, there have not been reported increases in cases of morbidity likely because of undiagnosed COVID-19 or mysterious deaths on the same scale as found in Asia, Europe, and America.

Similarly, every cadre of healthcare workers in this study recorded seropositivity, indicating that the exposure was widespread within the hospital environment. This is not unexpected because the study site is a tertiary hospital within a metropolis and members of staff come from different parts of the city where chances of community transmission are very high. In other places where community transmission has been documented, they were accompanied by staggering morbidity and mortality rates; this however was not the case in the African continent.^25^ The youthfulness of the African population, which has been identified as one of the protective factors against the disease severity, also comes to bear in this study because more than half of participants who were seropositive were between the age of 31–40 years.

A review of the distribution of seropositivity within different cadres of healthcare workers suggested that the professionals who have more contact or spend more time with patients are more likely to be seropositive; this pattern has previously been described.^26^ Doctors, nurses, and health assistants, in that order, recorded more seropositivity in this study than laboratory scientists/technicians and the nonmedical participants. This study also suggests that seropositivity within each department reflects the volume of patients attended to on a regular, basis with O&G, emergency, and medicine departments having higher seropositivity rates than microbiology, chemical pathology, and surgery departments. This also lends credence to a high possibility of community transmission; however, majority of them were asymptomatic and were silently infecting healthcare workers.^27^

There were limitations to this study. A larger sample size involving every department from the hospital would have been more reflective of the seroprevalence; however, the major clinical departments with significant patient flow were included in this study. More so, a quantitative assay of the SARS-CoV-2 IgG would have been more informative and offered important basis for comparison; this study essentially describes prevalence, for which a qualitative assay was considered sufficient.

In conclusion, seroprevalence of SARS-CoV-2 was high among the healthcare workers recruited for this study, although they were asymptomatic. It was not clear whether they got the infection from patients, colleagues, or the community where they reside, but chances of community spread appear to be considerably high, despite low disease severity, compared with the reports from European and American countries. This calls for more stringent precautionary measures among healthcare workers and provision of personal protective equipment, which has been reported to significantly lower SARS-CoV-2 positivity.^28^ These measures will hopefully help forestall further spread.
